# Barriers to help-seeking for memory problems in older adults

**DOI:** 10.1007/s41999-020-00371-6

**Published:** 2020-07-27

**Authors:** Ann Pearman

**Affiliations:** School of Psychology, Georgia Institute of Technology, 654 Cherry Street, J.S. Coon Building, Atlanta, GA 30332 USA

**Keywords:** Help-seeking, Barriers to care, Memory complaints, Hearing

## Abstract

**Purpose:**

Early detection of age- and disease-related cognitive problems affords patients the opportunities to receive medical treatment, engage in research, and plan for the future. Understanding help-seeking behavior has potential to aid both patients and clinicians. This study was designed to identify predictors of endorsed barriers to memory-related help-seeking as well as medical help-seeking endorsement.

**Methods:**

This cross-sectional correlational study used a convenience sample of 97 older adults. The participants answered anonymous questionnaires about subjective memory, mood, and health and several items designed to investigate help-seeking for memory issues.

**Results:**

Persons who endorsed multiple barriers to help-seeking were more likely to also endorse having hearing problems. In addition, participants who reported that they would not talk to a doctor or physician about memory concerns also had significantly worse subjective hearing.

**Conclusion:**

Hearing loss may be a particular risk for not seeking help for memory problems. Physicians and healthcare agencies can work to design outreach for persons who experience barriers, such as hearing loss and the concomitant outcomes.

## Key summary points

**Aim:**

The aim of this study was to investigate potential barriers to help-seeking for memory problems as well as outreach to providers.

**Findings:**

Participants who endorsed having hearing problems were the most likely to endorse barriers to help-seeking as well as speaking to a physician.

**Message:**

Physicians and healthcare agencies can work to design outreach for persons who experience barriers, particularly hearing loss.

## Introduction

As people live longer, there are going to be an increased number of older adults struggling with memory concerns and subsequent dementia [1]. Help-seeking for memory problems can lead to early diagnosis which can allow people to receive medical benefits and treatments, to emotionally prepare for future decline, and to build a care infrastructure to help them live in their homes as long as possible. However, if people feel concern about their memory but do not feel like they can talk to anyone about it or do not know what to do, this is important information in the development of outreach programs for both research and practice [2]. While there has been extensive work done examining predictors and correlates of memory complaints [3–6], only a few studies have specifically looked at help-seeking behavior for these concerns [2, 7–9]. Ramakers and colleagues [8] found that older adults who sought help at a memory clinic reported lower personal competence and a lower quality of life along with higher concern about developing dementia than the non-help-seekers. In a similar study examining help-seekers and non-help-seekers, it was shown that help-seekers were more likely to have a relative with dementia and to be more influenced by peer comparison [2]. While these studies do identify some differences between those groups who seek help and those who do not, they do not identify the perceived potential barriers to this type of help-seeking in people who have not sought help. The current study is designed to investigate possible barriers to action in terms of perceived memory problems in older adults.

Another question regarding help-seeking for memory problems, besides ideas about causation, is to whom the concerned individual might talk. For instance, are there people who would choose to not talk to their doctor? Begum and colleagues [7] qualitatively examined persons with subjective memory complaints and their help-seeking beliefs and attitudes regarding their primary care physicians. They interviewed formal help-seekers as well as those who did not seek formal help about their experiences and found that perceptions of PCPs differed between the two groups. The question remains which individual differences factors play into this evaluation.

A primary difference between this study and the several other help-seeking studies is that community-based nature of the sample. That is, the participants are not recruited from memory clinics. The current research was designed to be primarily descriptive in nature. Because of the novelty of the questions asked in this study, no predictions were made about the data.

## Methods

### Design

The study had a cross-sectional design and used anonymous self-report surveys.

### Participants

This convenience sample of older adult volunteers were recruited from senior centers and senior living facilities around Atlanta. Participants were given the packet of anonymous questionnaires to fill out at home as well as either a self-addressed stamped return envelope and/or access to a drop box in their facility. The study was approved as exempt by the Georgia State University Review Board (IRB) because of the anonymous nature of the data collection. The consent form was attached to the front of the packet. If participants decided to participate, they proceeded with the questionnaires.

### Survey questions

#### Demographic and health information

Participants answered basic demographic questions (e.g., age and education) and several questions about their current health status, including hearing, vision, and general health as well as number of doctor’s visits in the past year. For the subjective health questions, participants were asked to rate their hearing, vision, and overall health compared to their peers on a scale of 1 (poor) to 5 (excellent). Because there is a known effect of depression on memory complaints and help-seeking, participants were asked to fill out the 15-item Geriatric Depression Scale (GDS) [10]. The GDS is a self-report measure (yes/no to depression-related symptoms) specifically designed to avoid classification errors due to somatic symptoms. This version has been shown to be reliable and valid [11–13]. Scores can range from 0 to 15 with higher scores indicating more depression.

#### Subjective memory

Subjective memory was measured with two scales: the Memory Assessment Clinic-Complaint Questionnaire (MAC-Q: [14]) and the Memory Assessment Clinic-Global Memory Scale (MAC-G: [15]). Both of these scales are well validated and commonly used for assessment of memory concerns, particularly in clinical settings (e.g., [5, 16, 17]). The MAC-Q was designed to assess subjective memory change over the past 5 years. Participants answer six questions related to perceived change in several domains (e.g., How much has your memory for names changed in the past 5 years) and ranked their answers on a 1 (much worse now) to 5 (much better now). The MAC-G includes four items regarding overall memory (e.g., how would you describe your memory, on the whole, as compared to the best it is ever been?) which are rated on a 1 (much worse/poor) to 5 (much better/good. All items were reverse coded, so high scores indicate more perceived problems and decline. Because of the high degree of correlation between the items, the ten individual items for both of these scales were combined to form a subjective memory complaint (SMC) composite. This composite scale showed excellent internal reliability (*α* = 0.92).

In addition, participants rated how concerned they were about developing Alzheimer’s disease on a scale from 1 (not at all concerned) to 5 (very concerned) [18, 19].

#### Help-seeking variables

The specific variables of interest were (1) perceived barriers to help-seeking and (2) healthcare-related help-seeking actions. Barriers to help-seeking were measured with an 11-item checklist developed by the author where participants indicated whether a potential barrier (e.g., … I do not want to know if I have dementia) was relevant for them. Participants were encouraged to endorse as many barriers as they felt were applicable to themselves. Table 1 lists all of the barrier items. Healthcare-related help-seeking was measured with two potential healthcare points of contact that people may potentially talk about memory issues, specifically PCP and/or a specialist and a confidential memory screening. Participants could endorse one, both, or neither in terms of their willingness to help-seek if concerned about their own memory.

**Table 1:** Potential barrier items and frequencies of responses

I would not seek help for memory problems because	Absolute frequency (and percentage)
… there is nothing anyone can do to help memory problems	4 (4%)
… my memory is my own business and not anybody else’s	5 (5%)
… I am afraid I won’t be taken seriously	2 (2%)
… I believe memory changes are normal for people my age	9 (10%)
… it is difficult for me to find transportation	2 (2%)
… I am afraid of losing my insurance	0 (0%)
… I do not want to know if I have dementia	2 (2%)
… I would not know where to go	7 (8%)
… I do would not want anybody to know about them	4 (4%)
… I would be worried that I cannot afford that type of assessment	3 (3%)
… all of the places to do that are in a different part of town	1 (1%)

### Data analyses

Percentages, means, and standard deviations were used for the descriptive analyses to characterize the sample and the help-seeking variables. To simplify analyses, participants were given a score of 0 (no perceived barriers) or 1 (1 or more perceived barriers). Independent *t* tests were used to examine mean differences on the study variables between these two groups. To examine individual differences in outreach types, three distinct groups were formed: those who were willing to do a screening only, those who were willing to see a doctor only, and those who endorsed being willing to do both. Of note, there was only one individual who indicated an unwillingness to do either a screening or talk to a physician. That participant was excluded from this particular analyses. ANOVAs were conducted on all study variables using the three aforementioned groups.

## Results

### Respondent characteristics

Ninety-three volunteers participated in this study (*M* age = 75.36, SD = 7.49, range 58–95). Table 2 shows additional characteristics of the sample.

**Table 2:** Characterization of the sample studied

	Absolute frequency (and percentage) or mean (and standard deviation)
Age (in years)	75.36 (7.49)
Education (in years)	16.39 (2.87)
Sex	
Female	61 (66%)
Male	32 (34%)
Partnership status	
Married	53 (57%)
Widowed	23 (25%)
Other	17 (18%)
Neighborhood	
Urban	44 (47%)
Suburban	43 (46%)
Other	6 (7%)

### Potential barriers to help-seeking

The range of total acknowledged barriers endorsed by participants was 0–5 (out of a possible 11). The two most endorsed responses were “I would not seek help for memory problems because I would not know where to go” (10% of sample) and “I would not seek help for memory concerns because I believe that memory changes are normal for people my age” (7% of sample). Independent sample *t* tests were then computed to examine differences on study variables between the groups of people who did and did not acknowledge barriers (see Table 3). There were two significant differences between these two groups. The first was on subjective hearing with participants who endorsed one or more barriers reporting significantly worse self-rated hearing (*t* = 3.41, *p* = 0.001, *η*^2^ = 0.11). The second significant difference was on a subjective concern about AD question with participants who endorsed one or more barriers reporting significantly more concern about developing AD than those who endorsed no barriers (*t* = − 2.07, *p* = 0.04, *η*^2^ = 0.05).

**Table 3:** Perceived barrier group differences on health and memory

	*M* (SD)			*t* value
	Whole sample, *n* = 93	Perceived barriers		
		No, *n* = 19	Yes, *n* = 74	
GDS (range: 1–15)	1.378 (1.94)	1.23 (1.60)	1.89 (2.94)	–1.30**
Doctor visits past year	5.49 (6.80)	5.54 (7.14)	5.00 (5.29)	0.35**
Subjective health (ranges: 1–5)				
Vision	3.98 (.91)	4.04 (.80)	3.74 (1.19)	1.32**
Hearing	3.64 (1.04)	3.81 (.97)	2.94 (1.03)	3.41**
Overall health	3.87 (.92)	3.86 (.94)	3.89 (.81)	–0.14**
Subjective memory (ranges: 1–5)				
Subjective memory complaint	3.01 (.60)	2.98 (.63)	3.12 (.48)	–0.93**
Concern about developing AD	2.58 (1.04)	2.41 (1.21)	3.05 (1.13)	–2.07**

*M* mean, *SD* standard deviation, *GDS* 15-item Geriatric Depression Scale, *AD* Alzheimer’s disease

*p < 0.05; **p < 0.01

### Potential healthcare outreach if concerned about memory

**Fig. 1:**
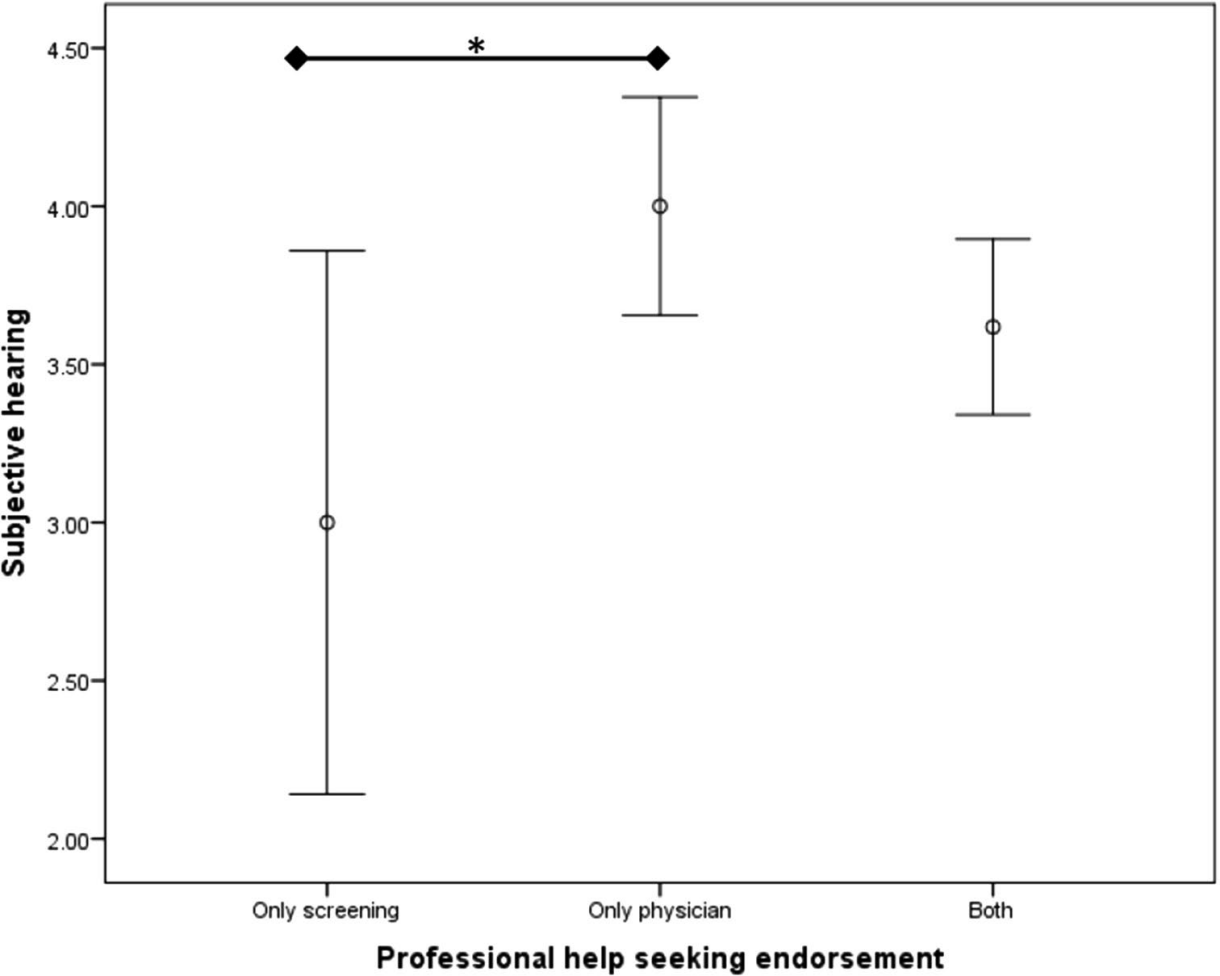
Professional help-seeking group differences on subjective hearing scores. Subjective hearing measured on 1–5 scale with higher scores indicating better hearing. Graph indicates mean value and 95% confidence intervals. Post hoc analyses were performed with the Fisher least significant difference test, *p < 0.05

In terms of healthcare outreach for perceived memory issues, Table 4 shows the frequency of endorsement and percentages. The only significant difference between the groups (screening only, physician only, and both) was on subjective hearing [*F*(2, 84) = 3.46, *p* = 0.04, *η*^2^ = 0.08]. Post hoc analyses using a Fishers least significant difference test revealed a significant difference only between participants who endorsed screening only versus physician only (see Fig. 1). These groups did not differ on any other variable.

**Table 4:** Potential help-seeking responses

If you noticed a memory problems would you …	Absolute frequency (and percentage)
Attend a free confidential memory screening ONLY	9 (10%)
Talk to your primary care physician or a specialist ONLY	23 (26%)
Both	55 (63%)
Neither	1 (1%)

## Discussion

Helping find ways for people with memory concerns to reach out and receive the care they need is an important goal for anyone who works with older adults and their families. This study is an important early step in starting to identify barriers and personal action plans for help-seeking for memory problems in older adults. The most commonly endorsed barrier to help-seeking for memory concerns was a lack of knowledge of where to go for help. This suggests that there is not always an obvious path to diagnosis or care for memory concerns for the general public. Professionals may assume that people know where to go while the patients, and perhaps their families, may be less than clear on action steps. This issue could potentially be even more challenging for people living in rural areas with fewer resources actually available. Messaging designed to guide people to the appropriate physicians, screening facilities, or memory clinics may be a way to help people. This is also true for research studies that are looking for diverse volunteers. If potential participants do not know where to go to find studies in which to enroll, then samples will end up being skewed toward the most educated and most informed.

Interestingly, many participants said they foresaw no barriers to help-seeking. However, the ones who did endorse one or more barriers were more likely to also endorse having poor hearing. In fact, subjective hearing came out as an important variable in both barrier endorsement and willingness to see a physician. These are interesting findings because while objective hearing loss has been cropping up in recent years as an important factor in cognitive decline [20] and potentially as related to both MCI to AD [21–23], subjective hearing loss has not been identified as a variable of interest in these areas.

An important place to start in deconstructing these findings is to actually explore the relationship between subjective and objective hearing. Obviously, in this anonymous self-report survey, there was not access to the participants measured hearing ability. However, several studies have shown a very high correlation between self-rated and objectively measured hearing loss [20, 24, 25] in that those who endorse poorer hearing are more likely to actually have objective deficits [20, 26]. In addition, given that the primary interest in the current study is in person’s perceptions of themselves and potential barriers to help-seeking, the use of self-rated hearing loss can still be considered valid. There may not be a one-to-one relationship between those who say they have hearing troubles and those who measure as having hearing troubles, but participant’s own perspective is important when asking about perceived barriers.

There are several possible reasons for the relationship between poor subjective hearing and endorsement of barriers. The first is that people with subjective hearing loss may have more difficulty communicating with their friends, family, and physicians, perhaps without even directly realizing they are having these troubles [21, 27, 28]. Communication may have become more difficult over time without a clear understanding of the reason for it. This type of communication deficit may lead to people to feel reticent about talking to others and may then compound their experience of barriers. There is also a literature on physician interaction regarding hearing loss and communication difficulties [29], which suggests that older adults with hearing issues often experience their physician appointments as particularly challenging [30, 31]. Seeking help for memory concerns may also lead to diagnosis of previously undiagnosed hearing impairments. While this may on the surface seem like a positive outcome, diagnosis of hearing impairment often leads to depression and anxiety due to self-perceptions and stereotype threat [32–35] which may be part of the reticence toward help-seeking from physicians. There is also the distinct possibility that the actual association between hearing loss and memory decline in later life may be driving this relationship [25, 36, 37]. While the lack of objective measures of hearing or memory does not allow us to further delve into this possibility, these findings open up an interesting potential avenue of inquiry for future studies as well as the development of outreach options.

A second goal of this study was to investigate potential healthcare-related actions for people if they were to encounter memory concerns. Most participants endorsed at least one healthcare access point where they could discuss memory issues. There was a group that said they would go to a confidential screening but would not speak to a physician about memory concerns. This suggests a reticence or barrier in professional help-seeking that may exist for many possible reasons, such as having a doctor know about a memory problem, a distrust of one’s doctor, or possibly even a lack of understanding of what the process may look like. In this study, the only significant difference between those who would see a doctor and those who would not was, again, subjective hearing. This suggests that there is a preference in those with poor subjective hearing to not interact with their primary care physicians, perhaps for the aforementioned reasons surrounding communication and vulnerability.

There are a couple of limitations of this study: The first is the subjective nature of the assessment without accompanying objective tests. That is, participants’ actual level of hearing loss and memory ability was not measured. In addition, the sample is fairly highly educated and may not be representative enough to make generalizations. However, as a preliminary foray into deterrents for memory help-seeking, it captures several very interesting potential research directions that have potential to impact how we approach outreach to elders with memory concerns. Future studies should include both hearing and memory tests as well as a deeper exploration as to the reasons behind barrier endorsement or lack of endorsement.

## Conclusion

This paper is an important first step in trying to understand why people may or may not seek help for memory concerns as well as who they might talk to about those concerns. Given the low endorsement of responses to barriers, an open-ended qualitative interview study may be needed to really try to understand barriers and perhaps causal attributions about those barriers and memory loss. Understanding potential barriers is an important task in creating outreach for all people with memory concerns. The finding of subjective hearing problems as related to all aspects of help-seeking in this study also suggests that gaining a better understanding of people’s experiences with the medical community when faced with communication challenges is important.
